# Factor structure and measurement invariance of the 8-item CES-D: a national longitudinal sample of Chinese adolescents

**DOI:** 10.1186/s12888-023-05316-4

**Published:** 2023-11-22

**Authors:** Shuxia Liu, Yuan Fang, Zhongyan Su, Jimin Cai, Zhiyan Chen

**Affiliations:** 1 CAS Key Laboratory of Mental Health, Institute of Psychology, Chinese Academy of Sciences, 16 Lincui Road, Chaoyang District, Beijing, 100101 China; 2 Department of Psychology, University of Chinese Academy of Sciences, Beijing, 101408 China

**Keywords:** Depression, Adolescents, Measurement invariance, CES-D 8, Longitudinal study

## Abstract

**Background:**

The 8-item Center for Epidemiologic Studies Depression Scale (CES-D 8) has been widely used to measure depressive symptoms in many large-scale surveys. Due to its brevity, it can lower costs, relieve respondent burdens, and ensure data quality. However, its factor structure and measurement invariance across gender and time among adolescents have not been adequately evaluated. This study investigated its factor structure and measurement invariance across gender and time among adolescents.

**Methods:**

The data was drawn from the China Family Panel Studies (CFPS) conducted in 2018 and 2020, with 3099 participants (46.82% girls) aged 11 to 18 in 2018. First, exploratory and confirmatory factor analyses were used to examine the factor structure of the CES-D 8. Next, multi-group confirmatory factor analysis was conducted to test its measurement invariance across gender and time. Finally, a longitudinal cross-gender test was conducted to further confirm the stability of the scale.

**Results:**

A two-factor structure was identified among the adolescents, including Negative Symptoms and Diminished Happiness Feeling. Measurement invariance across gender and time, as well as the longitudinal cross-gender invariance, was supported, with configural, factor loadings, thresholds and residual invariance.

**Conclusions:**

The factor structure of the CES-D 8 remains stable across gender and time among adolescents, indicating that it is a promising instrument for measuring depressive symptoms, especially in large-scale and longitudinal surveys.

**Supplementary Information:**

The online version contains supplementary material available at 10.1186/s12888-023-05316-4.

## Background

Depression is one of the leading causes of disability and death around the world, contributing greatly to the global health-related burden [1–3]. Furthermore, there is an increasing trend in the prevalence of depression globally in recent years [4], with the increase speed among adolescents exceeding that among adults [5, 6]. Given the fact that adolescents are experiencing dramatic developments in many areas such as social relationships, emotion and cognition, adolescent depression might result in subsequent detrimental outcomes. Therefore, it is significant to identify adolescents with relatively high depressive symptoms, thus to provide further diagnosis and targeted interventions as early as possible [7]. The present study focused on a screening instrument and tried to confirm its factor structure and stability across gender and time among adolescents, attempting to contribute to the universal screening of depressive symptoms.

### Depression in adolescents

Before puberty, depression is rare, but its prevalence increases rapidly from childhood to adolescence, especially in girls [8]. Previous literature has suggested that it is highly associated with adverse developmental outcomes in later life, including (1) approximately 50% of adolescents with depression suffering depression or anxiety disorders in adulthood [9]; (2) higher risk of other mental illness or risk/criminal behaviors [10, 11]; (3) elevated probability of poor physical health and incompetency as adults [9].

Nevertheless, the long-term outcomes differ among individuals. For instance, a study revealed that the association between adolescent depression and poor mental health in adulthood might depend on the persistence or severity of the symptoms during adolescence [12]. Another prospective research found that adult psychiatric and functional outcomes were associated with cumulative exposure to depression, including the number of episodes and the average degree [13]. In addition, receiving community care or professional mental health services appeared to improve outcomes in later life [10]. Considering the above findings, it is urgent to screen and identify depressive symptoms among adolescents in order to provide effective and timely interventions [14, 15].

### Measurements of depressive symptoms

Many scales have been used to screen and discern depressive symptoms in large populations. According to previous literature, there is no evidence that one measure is better than the others, and the choice may depend on numerous considerations [7, 16, 17]. For instance, Beck Depression Inventory (BDI) may be a more accurate measure of mild or “neurotic” depressions [16, 18], Patient Health Questionnaire-9 (PHQ-9) may be used to diagnose depressive symptoms and evaluate their severity [17], while the Center for Epidemiologic Studies Depression Scale (CES-D) is appropriate for measuring depressive symptoms in the general population [19]. Considering the purpose of screening depressive symptoms among general adolescents, this study focused on the CES-D.

The original CES-D consisted of 20 items, measuring four factors including “depressed affect”, “positive affect”, “somatic and retarded activity” and “interpersonal” [19]. The scale was widely used in the Chinese context and showed adequate reliability and validity [20, 21]. However, it was time-consuming and burdensome for respondents in large-scale social surveys, so a short 8-item CES-D (CES-D 8) [22] was proposed to suit such surveys. It had been adopted in many large-scale surveys, such as Asset and Health Dynamics Among the Oldest Old (AHEAD) [23], Health and Retirement Study (HRS) [24] and European Social Survey (ESS) [25].

Although the CES-D 8 has been widely used, different factor models have been identified in previous literature. For instance, a two-factor model was supported in American samples, including “depressed mood” and “somatic complaints” [22, 24]. A different two-factor model was found in South Africa residents, with negative and positive items loading on “negative affect” and “diminished positive affect” respectively [26]. Additionally, a one-factor model with correlated uniqueness between two positively worded items was revealed using samples of Europeans [25, 27]. Therefore, the dimensions of the CES-D 8 appeared to be associated with cultural differences.

Moreover, most of the research above involved general population or aged adults. The latter might have energy difficulty in completing a time-consuming survey [25]. Adolescents, however, susceptible to reduced sustained attention due to “decreased motor control and increased impulsivity” [28], will also benefit from an effective and efficient instrument. Nevertheless, there have been few such studies focusing on adolescents. Together, further research is required to examine the factor structure of the CES-D 8 among adolescents in different countries. Therefore, the first goal of our study is to examine the factor structure of the CES-D 8 among adolescents based on a Chinese sample.

### Gender differences in adolescent depression

Gender differences in depression (i.e., females are more likely to suffer major depressive disorder than males) might be one of the most robust conclusions in psychopathology studies [29]. It emerged from puberty and peaked at the age of 15 to 18 [30, 31]. Although many research suggested there were still gender differences when adolescents entered their young adulthood [31, 32], some studies revealed that the differences were becoming narrow and even disappeared during the developmental period [33, 34]. The mixed results implied more studies required to further explore the gender differences.

Most research has focused on the association between gender and depressive symptoms, ignoring the fact that assessments of depression per se can introduce bias. That is, measurement bias between gender may influence the examination of difference and its magnitude [35]. A study of gender differences in depression found that eliminating measurement bias sometimes resulted in different conclusions [36]. Therefore, it is necessary to test measurement invariance for an effective comparison of depression across gender. If the comparison is conducted based on latent means, scalar measurement invariance is required, otherwise, the difference across group may reflect the systematic response bias [35]. Similarly, if the comparison is conducted based on manifest means, strict measurement invariance is required, otherwise, the credibility of interpretations of the results will be undermined [37].

Targeting the CES-D 8, the examination of measurement invariance across gender showed inconsistency in previous literature. Some studies revealed strict measurement invariance across gender [25, 38], while others found partial measurement invariance [26, 36]. Furthermore, the samples in these studies were all adults, lack of empirical investigations into adolescents. To conclude, it is worthwhile to conduct a measurement invariance test across gender among adolescents. Therefore, our second goal is to examine the measurement invariance across gender among adolescents.

### Depression development in adolescence

As mentioned previously, the rapid rise in the prevalence of depression occurs in adolescence, with subsequent development leading to different outcomes; therefore, it is essential to conduct longitudinal analyses to better understand its development over time. Researchers have been devoted to exploring the different developmental trajectories of depressive symptoms and the associated risk and protective factors from early adolescence to young adulthood, in order to develop more targeted strategies for prevention and intervention [39, 40].

However, the same instrument may measure different constructs of depressive symptoms at different time points throughout adolescence, as adolescents are experiencing dramatic changes in thinking modes, self-conception, social cognition and interpersonal relationships, which can affect how they feel and report their depressed mood [41]. Nonetheless, measurement invariance over time was seldom mentioned in the previous longitudinal studies. Longitudinal analyses without measurement invariance examination are not tenable, for it is unable to judge whether observed changes are caused by the development of the construct of interest or measurement bias. Therefore, it is essential to examine measurement invariance over time prior to longitudinal analyses.

Specifically, few studies have examined the measurement invariance of the CES-D 8 over time, as well as the longitudinal cross-gender invariance (i.e., both cross-gender and longitudinal measurement invariance are simultaneously tested) [42]. Without such examinations prior to longitudinal analyses, the results may not hold themselves and bias subsequent meta-analyses. Thus, our third goal is to assess measurement invariance of the CES-D 8 over time among adolescents, followed by a longitudinal cross-gender measurement invariance test.

### The present study

This study focuses on the validity examination of the CES-D 8, investigating its factor structure and measurement invariance across gender and time, aiming to (1) confirm whether it is suitable for adolescents; (2) provide empirical evidence to discriminate the true different depressive symptoms from just measurement bias caused by gender and/or time [35]. Furthermore, the application of the brief scale will contribute to (1) easier and more efficient survey in a large scale to screen out the adolescents with relatively high depressive symptoms at a lower cost; (2) relieving the adolescents’ respondent burden and optimizing their motivation to ensure the data quality [43].

To achieve the above goals, this study was performed as follows. First, considering the absence of related research on the factor structure of the CES-D 8 among adolescents, the factor structure was identified using exploratory factor analysis (EFA) and confirmatory factor analysis (CFA). Second, the obtained factor structure was used to test measurement invariance across gender. Third, the measurement invariance over time and the longitudinal cross-gender invariance were examined to provide evidence for longitudinal studies.

## Methods

### Participants and procedure

The participants came from the China Family Panel Studies (CFPS), which was a nationally representative social survey, conducted by the Institute of Social Science Survey (ISSS) of Peking University [44]. The CFPS employed a multi-stage probability sampling method, extracted by the means of implicit stratification, including information on levels of community, family and individual [44]. For the general-purpose, the data was collected on a household basis, covering 94.5% of the population, who were from 25 provinces (or their administrative equivalents) in Chinese mainland [44].

The survey was conducted every two years since 2010. This research chose the data in 2018 (T1) and 2020 (T2), with the participants aged between 11 and 18 in 2018. There were 3315 adolescents taking part in the survey at T1, and 216 of them did not respond to any of the items in the instrument (reported in the next section). After removing their data, 3099 adolescents (46.82% girls) remained, whose average age was 14.31 (*SD* = 2.28). 2663 (85.93%) of these adolescents were Han nationality, 423 (13.65%) were non-Han nationality, and 13 (0.42%) did not report their nationality. 1768 (57.05%) adolescents lived in rural areas, 1307 (42.17%) lived in urban areas, and 24 (0.77%) did not report the residence. The average family income (log transformed) was 4.74 (0.41). At T2, there were 1978 adolescents (48.08% girls) filled out the questionnaire, and their average age was 16.36 (*SD* = 2.30). Among them, 1711 (86.50%) adolescents were Han nationality, 263 (13.30%) were non-Han nationality, and 4 (0.20%) did not report their nationality. 1141 (57.84%) adolescents lived in rural areas, 816 (41.25%) lived in urban areas, and 18 (0.91%) did not report the residence. The average family income (log transformed) was 4.74 (0.42).^1^

Attrition analyses indicated that the participants who retained or dropped out at T2 did not differ significantly in gender (*χ*^*2*^(1) = 3.47, *p* = 0.062), age (*t*(3097) = 0.27, *p* = 0.789), nationality (*χ*^*2*^(1) = 0.68, *p* = 0.409), residence (*χ*^*2*^(1) = 1.68, *p* = 0.195) and family income (*t*(3068) = -0.04, *p* = 0.971).

In this study, the survey at T1 was launched in June 2018 and completed in May of the following year. The survey was conducted by telephone or face-to-to face conversations using computer-assisted personal interviews. Among the participants, 2606 adolescents (84.09%) were interviewed face-to-face. The survey at T2 was conducted in the same way between September and December in 2020; nevertheless, only 240 participants (12.13%) were interviewed on the spot because of the COVID-19 pandemic. All the adolescents responded by themselves.

### Measures

#### Depressive symptoms

The CES-D 8 was used to measure depressive symptoms [22]. The participants were asked how often they experienced some mental state in the past week, with a 4-point rating scale, ranging from 1 (Never, less than one day) to 4 (most of the time, 5–7 days). 2 of the 8 items (i.e., “feel happy” and “have a happy life”) were reverse-coded prior to data analysis. The internal consistency reliability of the instrument was assessed by omega (ω) coefficient, with the results ω_T1_ = 0.71, ω_T2_ = 0.76.

### Data analysis

Four steps were used to investigate the factor structure and measurement invariance of the CES-D 8 among the adolescents. First, EFA was performed on the data at T1 to identify the factor structure of the CES-D 8. Exploratory structural equation modeling (ESEM) was used because it could handle EFA with correlated residuals [46, 47]. Parallel analysis indicated that two factors should be retained (See Table S1 in the online supplements), so four models were examined, including one to two-factor ESEM models, with and without accounting for correlated residual between two reverse-coded items. Of particular note was that the one-factor model with correlated residual was Karim’s [25] and Van de Velde’s [27] model. Oblique rotation with GEOMIN strategy was used to obtain the ultimate factor loadings.

**Fig. 1:**
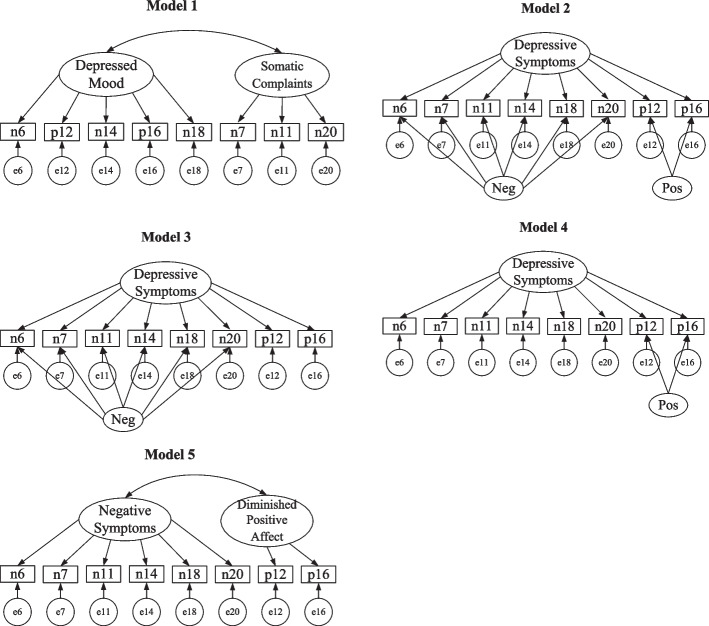
Five structural equation models of CES-D 8 for the data at T2. Model 1 = Steffick’s and Turvey’s two-factor model; Model 2 = bi-factor model with one general factor and two specific factors measuring positive and negative method effects respectively; Model 3 = bi-factor model with one general factor and one specific factor measuring negative method effect; Model 4 = bi-factor model with one general factor and one specific factor measuring positive method effect; Model 5 = the two-factor model uncovered by the EFA

Second, using the data at T2, CFAs were performed to compare the factor structure found by the EFA with several competing models to select the final factor structure. The competing models included (1) Turvey’s [22] and Steffick’s [24] two-factor model (Model 1 in Fig. 1), (2) three correlated trait-correlated method (CTCM) models (Model 2–4 in Fig. 1), using latent method factor(s) to represent wording effects [48–51]. Specifically, Model 1 to Model 4 were also examined using the data at T1 (see Tables S2 and S3 in the online supplements).

Third, following the guidelines by Millsap and Yun-Tein [52], the measurement invariance across gender was tested using the multi-group CFA (MG-CFA) based on the best fitting model from the previous CFAs. A series of models were examined, including (1) a configural invariance model where each factor was constrained to have the same indicators across groups; (2) a metric invariance (weak invariance) model where the factor loadings were constrained to be equal across groups; (3) a threshold invariance model where the thresholds of each indicator were constrained to be equal across groups, which paralleled the scalar invariance (strong invariance) model for continuous indicators (here the items were considered as ordinal indicators); (4) a residual invariance (strict invariance) model additionally constraining equivalent residual variance across groups. These four models were hierarchical and the adjacent pairs were statistically compared to examine the measurement invariance.

Fourth, the measurement invariance over time and longitudinal cross-gender invariance was examined. The former used the guidelines by Liu et al. [53], while the latter used the joint guidelines by Millsap and Yun-Tein [52] and Liu et al. [53], referring to [42, 54] meanwhile. The details of the longitudinal cross-gender test were as follows. In the baseline model, (1) the test was performed among four groups (2 genders × 2 occasions), one of which was set up as reference group; (2) the factor loadings were freely estimated, except that the factor loading of the first indicator of each factor was set to 1; (3) the thresholds were freely estimated, except that the subsets which were constrained to be invariant across groups (i.e., one threshold for each item and a second threshold for the marker variable) [53]; (4) the factor means were freely estimated, except that the factor means were constrained to 0 in the reference group; (5) the residual variances were freely estimated, except that the residual variances were constrained to 1 in the reference group; (6) the residuals of the same items were not associated across gender groups, but associated at different time points. The constraints of the other longitudinal cross-gender invariance models could be imposed referring to the previous paragraph.

The EFA, CFAs and MG-CFAs were conducted with Mplus 7.4 [47], except that parallel analysis were conducted by R (version 4.2.1), using the package *psych* (version 2.2.5) [55, 56]. As depressive symptoms were rated on a 4-point Likert scale, the items were considered categorical indicators [57]. The mean- and variance-adjusted diagonal weighted least squares (WLSMV) estimator was used in the analyses according to the software manual and recent literature [20, 58, 59]. As for missing values, pairwise deletion was used by default due to the use of the WLSMV estimator and the absence of external model covariates.

Multiple criteria were considered in order to evaluate the model fit. For the EFA and CFAs, the chi-square (χ^2^) statistics, comparative fit index (CFI), Tucker-Lewis index (TLI) and root mean square error of approximation (RMSEA) were reported. The goodness of fit was assessed by the following combination of multiple criteria, with CFI and TLI > 0.95 and RMSEA < 0.06 for a relatively good fit [60]. As for the χ^2^ statistics, they were presented here only for their use in calculating RMSEAs, not for evaluation of the model fit because they were sensitive to sample size [59].

To evaluate the measurement invariance, the changes of several indices were presented, consisting of changes in chi-square statistics (Δχ^2^), comparative fit index (ΔCFI), and root mean square error of approximation (ΔRMSEA). ΔCFI < 0.01 and ΔRMSEA < 0.015 indicated measurement invariance [61, 62].

## Results

### Exploratory factor analysis

Table 1 showed the model fit indices of three models, including one and two-factor model without correlated residual and one-factor model with correlated residual. The two-factor model with correlated residual was not suitable for the data because the residual covariance matrix was not positive definite, so its model fit indices were not provided. One-factor model without correlated residual yielded poor model fit. Both one-factor model with correlated residual and two-factor model without correlated residual had adequate model fit, with their estimated model parameters provided in Table 2.

**Table 1:** Model fit indices in the exploratory factor analysis

EFA Model	*χ*^*2*^	*df*	*χ*^*2*^/*df*	CFI	TLI	RMSEA (90% CI)
1-factor without correlated residual	2284.910^***^	20	114.246	0.772	0.681	0.191 (0.185 0.198)
1-factor with correlated residual	122.156^***^	19	6.429	0.990	0.985	0.042 (0.035 0.049)
2-factor without correlated residual	132.804^***^	13	10.216	0.988	0.974	0.055 (0.046 0.063)

^***^p < 0.001

**Table 2:** The estimated model parameters of one-factor model with correlated residual and two-factor model without correlated residual

Item number	Item content	1-factor with correlated residual	2-factor without correlated residual	
		DS	NS	DHF
6	I am in a low spirit	**0.75**	**0.76**	-0.02
7	I find it difficult to do anything	**0.63**	**0.65**	-0.04
11	I cannot sleep well	**0.53**	**0.53**	0.01
12	I feel happy	0.28	-0.01	**0.88**
14	I feel lonely	**0.78**	**0.76**	0.04
16	I have a happy life	0.32	0.09	**0.70**
18	I feel sad	**0.79**	**0.79**	0.00
20	I feel that I cannot continue with my life	**0.70**	**0.67**	0.07
Variance^a^		39.30%	52.66%	

^a^The content in the last row demonstrated explained percentage of the factor(s) in each model to the total variance

*DS* Depressive Symptoms, *NS* Negative Symptoms, *DHF* Diminished Happiness Feeling

In the one-factor model with correlated residual, the factor loadings of the two reverse-coded items were only 0.28 and 0.32, which were both below the cut-off value of 0.40 recommended by Worthington and Whittaker [63]. In addition, the model only explained 39.30% of the total variation in the sample. On the other hand, the two-factor model without correlated residual had no cross-loading items, with factor loadings ranging from 0.53 to 0.88, all above the cut-off value of 0.40. Furthermore, it explained 52.66% of the total variation in the sample. Hence, the two-factor model without correlated residual (Model 5) was selected and used in the subsequent analyses.

In Model 5, the first factor, named Negative Symptoms, consisted of depressed affects (sad, low spirit, lonely, cannot continue) and somatic complains (difficult to do, sleep not well). With an eigenvalue of 2.940, it explained 36.75% of the total variation in the sample. The second factor, named Diminished Happiness Feeling, included two reverse-coded items relating to happiness feeling. With an eigenvalue of 1.273, it explained 15.91% of the total variation in the sample. The reliability coefficient for the Negative Symptoms factor was 0.74 (omega coefficient) and the coefficient for the Diminished Happiness Feeling factor was 0.71 (Spearman-Brown coefficient) [64].

### Confirmatory factor analysis

The results of the CFAs were shown in Tables 3 and 4. Model 1 did not provide adequate fit at all. Model 2 considered both positive and negative wording effects, however, it could not be properly identified, which was consistent with previous literature [65]. Model 4 and Model 5 were equivalent models. The Diminished Happiness Feeling factor in Model 5 was replaced with the specification that two positive items loaded simultaneously on the substantial factor and method factor in Model 4 [66]. Although Model 3, Model 4 and Model 5 demonstrated comparable fit and explained variance, the convergent validity of the substantive factor in Model 3 and Model 4, measured by Average variance extracted (AVE), was below the cut-off value 0.5 [67]. For Model 5, the AVEs of the two factors were both above the cut-off value, indicating good convergent validity. In the meanwhile, the correlation coefficient between the two factors in Model 5 was 0.40, whose square was much lower than the AVEs, demonstrating high discriminant validity. Therefore, from the statistical point of view, Model 5 could be selected as the most appropriate model.

**Table 3:** Fit indices of the competing models using the data at T2

Model	*χ*^*2*^	*df*	*χ*^*2*^/*df*	CFI	TLI	RMSEA (90% CI)
Model 1	1636.194^***^	19	86.115	0.826	0.743	0.207 (0.199 0.216)
Model 2^a^	—	—	—	—	—	—
Model 3	61.645^***^	14	4.403	0.995	0.990	0.041 (0.031 0.052)
Model 4	60.221^***^	19	3.170	0.996	0.993	0.033 (0.024 0.043)
Model 5	60.220^***^	19	3.169	0.996	0.993	0.033 (0.024 0.043)

^a^Model 2 could not be properly identified when applied to the data at T2

^***^p < 0.001

**Table 4:** Factor loadings of the competing models using the data at T2

Item	Model 1		Model 3		Model 4		Model 5	
	DM	SC	DS	Neg	DS	Pos	NS	DHF
n6	0.74		0.27	0.72	0.76		0.76	
n7		0.78	0.29	0.70	0.75		0.75	
n11		0.61	0.28	0.54	0.61		0.61	
n14	0.77		0.34	0.71	0.79		0.79	
n18	0.78		0.31	0.74	0.80		0.80	
n20		0.75	0.30	0.68	0.74		0.74	
p12	0.59		0.80		0.32	0.74		0.80
p16	0.61		0.82		0.33	0.74		0.82
AVE	0.49	0.51	0.23	0.47	0.44	0.55	0.55	0.66
Variance^a^	50.15%		58.30%		57.88%		57.95%	

^a^The content in the last row demonstrated explained percentage of the factor(s) in each model to the total variance

*DM* Depressed Mood, *SC* Somatic Complaints, *DS* Depressive Symptoms, *Neg* Negative Method, *Pos* Positive Method, *NS* Negative Symptoms, *DHF* Diminished Happiness Feeling, *AVE* Average variance extracted

From the substantive point of view, Model 5 suggested two substantive components, including Negative Symptoms factor and Diminished Happiness Feeling factor, which was preferred because (1) in the original article, Radloff argued that the positive items were used to break tendencies toward response set and evaluate positive affect [19]; (2) with depression, both the World Health Organization (WHO) and American Psychiatric Association (APA) considered that it involved depressed mood or loss of interest or pleasure, implying that diminished positive emotions was not just a wording effect but an important dimension [1, 68]; (3) in a broader perspective, the WHO noted that, “Mental health is an integral component of health and well-being and is more than the absence of mental disorder” [69]. In line with this, a dual-factor model of mental health including associated positive and negative factors was recommended to better explain mental health [70, 71].

Based on the above analysis, Model 5 was eventually selected and used in the subsequent measurement invariance test.

### Measurement invariance

The model fit indices of the MG-CFAs were presented in Table 5. In the measurement invariance test across gender, all models in the hierarchy fitted well at both waves. The results showed that the model fit was not significantly deteriorated while imposing more and more strict constraints (including factor loadings, thresholds and residual variance) across groups, suggesting that the CES-D 8 measured the same construct for males and females at two time points.

**Table 5:** Model fit indices of the measurement invariance tests

model	*χ*^*2*^	*df*	*χ*^*2*^/*df*	*Δχ*^*2*^	RMSEA (90% CI)	CFI	TLI	ΔRMSEA	ΔCFI
Invariance test across gender at T1 (*N* = 3099, Male = 1648, Female = 1451)									
configural	145.374^***^	38	3.826		0.043 (0.035 0.050)	0.990	0.985		
metric	159.739^***^	44	3.630	16.371^*^	0.041 (0.034 0.048)	0.989	0.986	-0.001	-0.001
threshold	163.872^***^	58	2.825	19.229	0.034 (0.028 0.041)	0.990	0.990	-0.007	0.001
strict	238.184^***^	66	3.609	69.251^***^	0.041 (0.035 0.047)	0.983	0.986	0.007	-0.007
Invariance test across gender at T2 (*N* = 1978, Male = 1027, Female = 951)									
configural	76.472^***^	38	2.012		0.032 (0.021 0.042)	0.996	0.994		
metric	82.350^***^	44	1.872	6.989	0.030 (0.020 0.040)	0.996	0.995	-0.002	0.000
threshold	86.099^**^	58	1.484	11.853	0.022 (0.011 0.032)	0.997	0.997	-0.008	0.001
strict	124.283^***^	66	1.883	36.620^***^	0.030 (0.022 0.038)	0.994	0.995	0.008	-0.003
Invariance test over time (T1: *N* = 3099; T2: *N* = 1978)									
configural	191.674^***^	90	2.130		0.019 (0.015 0.023)	0.994	0.993		
metric	205.933^***^	96	2.145	15.016^*^	0.019 (0.016 0.023)	0.994	0.993	0.000	0.000
threshold	240.565^***^	110	2.187	37.118^***^	0.020 (0.016 0.023)	0.993	0.992	0.001	-0.001
strict	289.206^***^	118	2.451	49.229^***^	0.022 (0.018 0.025)	0.991	0.991	0.002	-0.002
Longitudinal cross-gender invariance test (T1: *N* = 3099, Male = 1648, Female = 1451; T2: *N* = 1978, Male = 1027, Female = 951)									
configural	322.486^***^	190	1.697		0.021 (0.017 0.025)	0.993	0.991		
metric	346.217^***^	208	1.665	27.102^†^	0.021 (0.017 0.024)	0.993	0.991	0.000	0.000
threshold	414.599^***^	250	1.658	75.547^**^	0.021 (0.017 0.024)	0.991	0.992	0.000	-0.002
strict	532.547^***^	266	2.002	122.085^***^	0.025 (0.022 0.029)	0.986	0.987	0.004	-0.005

^†^*p* < 0.1. ^*^p < 0.05. ^**^p < 0.01. ^***^p < 0.001

In the longitudinal measurement invariance test, the changes in CFI and RMSEA indicated that strict invariance was supported over a two-year period. Moreover, the longitudinal cross-gender invariance was supported, demonstrating that the scale measured the same construct across gender over a two-year period. The structural invariance and actual gender differences and temporal differences in the CES-D 8 factor scores were also examined, and the results were provided in Tables S4 and S5 of the supplements.

## Discussion

The current study aimed to provide more empirical evidence on the psychometric properties of the CES-D 8. A sample from the CFPS was used to identify the factor structure of the CES-D 8 among adolescents and examine its measurement invariance across gender and time (a two-year period). Previous literature focused primarily on general and aged adults, while few studies examined the factor structure and measurement invariance across gender and time among adolescents, especially in such a national sample. The study had three important findings.

First, based on the EFA and CFAs, a two-factor model was identified, including Negative Symptoms and Diminished Happiness Feeling. The Diminished Happiness Feeling factor contained two reverse-coded items describing happiness affect and the Negative Symptoms factor contained the other six items. The factor structure was similar to the previous results in Irish and South Africans [26, 72]. Although Adams et al. [26] made a slight modification to the two items, all the items ultimately loaded on “Negative Affect” factor and “Diminished Positive Affect” factor respectively. The Negative Symptoms factor in our model involved items from the “somatic complains” and “depressed affect” factors in Radloff’s [19] original structure, and such integration had been observed in the studies conducted among Asians, Europeans and Africans, suggesting that depression might be characterized by some inherent mental and physical experiences across ages and cultures [25, 26]. It should also be noted that the two-factor model in current study was different from Karim’s and Van de Velde’s model in the two reverse-coded items, although they were equivalent models. That might be because their participants were aged adults, and anhedonia/loss of interest was more common in aged adults than adolescents [73, 74].

The results were inconsistent with those found in American samples [22, 24], where a different two-factor model was identified, including “depressed mood” and “somatic complaints” (i.e., Model 1). The most common explanation for this inconsistency was that Chinese people were more ashamed of reporting mental illness than westerners [75]. However, the same integration of “depressed mood” and “somatic complaints” was also found among the Europeans [25]. There might exist other explanations, such as generation gap, as the American participants were significantly older than the European participants, or measurement bias, as dichotomous variables were used in the American studies while 4-categorical variables were used in the European studies. In summary, it is noteworthy that the construct of the depressive symptoms among the Chinese adolescents was not totally same with the Europeans and the Americans. More research was required to confirm the factor structure of the CES-D 8 among populations at different ages and varied cultures.

Second, based on our two-factor model, strict invariance across gender was supported, indicating that the construct (depressive symptoms) measured by the CES-D 8 was reliable, and the latent means and manifest means could be compared meaningly between girls and boys. This finding was consistent with the previous literature involving measurement invariance across gender, although their participants were young adults or aged adults, which might suggest that the CES-D 8 had comparable cross-gender stability across age groups [25, 27, 38].

Third, the longitudinal measurement invariance test suggested that strict invariance was supported in a two-year period among adolescents, even across gender. To our knowledge, although the CES-D 8 has been applied in longitudinal studies, this is the first research on longitudinal properties of the scale, especially across gender simultaneously [76]. Therefore, our findings of longitudinal strict invariance of the CES-D 8 extends its utility in terms of the longitudinal research.

Put it all together, the CES-D 8 is a suitable instrument for measuring depressive symptoms among adolescents. The brevity makes it preferable for large-scale administration to screen out the adolescents with relatively high depressive symptoms at a lower cost; in the meanwhile, it can guarantee a relatively robust data quality since it relieves the respondent burden due to adolescents’ lack of attention [28, 43]. Furthermore, the measurement invariance test provided empirical evidence for the stability of the scale among adolescents, implicating the meaningful comparisons across gender or true changes in the development of depressive symptoms.

In spite of the strengths, there are four limitations in this study. First, the findings are based on an exclusive Chinese adolescent sample, so the generalizability of the CES-D 8 was not examined. Racial/ethnic generalizability is critical to any of the psychiatry measures [58]. In aged adults, different factor structures of the CES-D 8 had been found among Americans, Europeans and Africans [22, 24–27]. However, little research has been conducted to examine the psychometric properties of the scale in adolescents. More research should be conducted among diverse cohorts in different cultures in order to reach a more pervasive conclusion.

Second, despite the longitudinal design of the current study, data was only collected at two waves over a two-year period. In future research, more data at three waves or more over a longer period should be obtained. Using these data, not only the stability of the CES-D 8 can be examined more deeply, but also a latent growth model (LGM) can be established [77].The LGM can describe the developmental trajectories of the depressive symptoms over time, identify the intra-individual and inter-individual variability in reference levels and trajectories, and examine the different contributions of some protective and risk factors to the reference levels and trajectories.

Third, the CES-D 8 was self-reported and the only instrument used to measure depressive symptoms in the current study. It would be more reliable if interviews and/or other-reported instruments are combined. Furthermore, reliable interview instruments, such as WHO-Composite International Diagnostic Interview (CIDI), can be treated as a temporary “gold standard”, allowing analysis of the performance of the CES-D 8 [78, 79]. The performance includes its sensitivity (ability to correctly identify patients), specificity (ability to correctly identify non-patients) and receiver operating characteristics (ROC) curves (used to establish an appropriate cut-off value to distinguish patients from non-patients) [80].

Fourth, although the possible common method bias caused by the wording effects had been considered in the EFA and CFA, the Diminished Happiness Feeling factor of the final model was the mix of substantial and method components. It would be more reliable if additional variables are introduced into the research and permit more advanced methods to identify and control method bias, such as confirmatory factor analysis marker technique and IV (i.e., independent variable) technique [81, 82].

## Conclusions

This study reveals that the CES-D 8 remains reliable and stable across gender and over a two-year period among adolescents. The findings extend the related literature from general population or aged adults to adolescents, and from cross-sectional designs to longitudinal ones, indicating that it is a promising instrument to screen depressive symptoms among adolescents, especially in large-scale and longitudinal surveys.

### Supplementary Information

**Additional file 1: Table S1.** Parallel analysis of the data at T1. **Table S2.** Fit indices of the competing models using the data at T1. **Table S3.** Factor loadings of the competing models using the data at T1. **Table S4.** Model fit indices of the structural invariance tests. **Table S5.** actual gender and temporal differences in the CES-D 8 factor scores.

## Data Availability

The datasets generated and/or analyzed during the current study are available in the China Family Panel Studies repository, http://www.isss.pku.edu.cn/cfps/. The codes were available at OSF HOME, https://osf.io/e9jqg/.

## Abbreviations

CES-D: Center for Epidemiologic Studies Depression Scale
CFPS: China Family Panel Studies
BDI: Beck Depression Inventory
PHQ-9: Patient Health Questionnaire-9
AHEAD: Asset and Health Dynamics Among the Oldest Old
HRS: Health and Retirement Study
ESS: European Social Survey
EFA: Exploratory factor analysis
CFA: Confirmatory factor analysis
ISSS: Institute of Social Science Survey
MG-CFA: Multi-group CFA
WLSMV: Mean- and variance-adjusted diagonal weighted least squares
CFI: Comparative fit index
TLI: Tucker-Lewis index
RMSEA: Root mean square error of approximation
IV: Independent variable
AVE: Average variance extracted

## Footnotes

1 Except family annual income, the other demographic covariates were drawn from the cross-year library of the individual core variables released with the data at T1. The variables in the cross-year library were considered more reliable than ones from a single survey, for the data cleaning personnel compared them from previous surveys and selected the most reasonable values according to certain principles [45].
