# Supplementary material for: Factor structure and measurement invariance of the 8-item CES-D: a national longitudinal sample of Chinese adolescents
Source: BMC Psychiatry. 2023 Nov 22;23:868. doi: 10.1186/s12888-023-05316-4 (PMC10664487; doi:10.1186/s12888-023-05316-4)
Supplement: Supplementary file 1 — Additional file 1: Table S1. Parallel analysis of the data at T1. Table S2. Fit indices of the competing models using the data at T1. Table S3. Factor loadings of the competing models using the data at T1. Table S4. Model fit indices of the structural invariance tests. Table S5. actual gender and temporal differences in the CES-D 8 factor scores. [file 12888_2023_5316_MOESM1_ESM.docx]

**Supplemental Materials for:**

**Factor structure and measurement invariance of the 8-item CES-D: a national longitudinal sample of Chinese adolescents**

**Table S1** Parallel analysis of the data at T1

|  | Principal axis factor analysis | | Principal components analysis^a^ | |
| --- | --- | --- | --- | --- |
| Eigenvalues | real data | simulated data | real data | simulated data |
| 1 | 3.153 | 0.399 | 3.686 | 1.075 |
| 2 | 0.560 | 0.248 | 1.386 | 1.048 |
| 3 | 0.143 | 0.178 | 0.732 | 1.029 |
| 4 | 0.031 | 0.129 | 0.603 | 1.009 |
| 5 | -0.012 | 0.077 | 0.506 | 0.994 |
| 6 | -0.070 | 0.046 | 0.399 | 0.972 |
| 7 | -0.140 | 0.010 | 0.355 | 0.949 |
| 8 | -0.507 | -0.017 | 0.336 | 0.925 |

Note.

^a^ The eigenvalues based on principal components analysis were the same with the eigenvalues in Mplus.

**Table S2** Fit indices of the competing models using the data at T1

| Model | *χ²* | *df* | *χ²*/*df* | CFI | TLI | RMSEA (90% CI) |
| --- | --- | --- | --- | --- | --- | --- |
| Model 1 | 2225.279^***^ | 19 | 117.120 | 0.778 | 0.673 | 0.194 (0.187 0.200) |
| Model 2^a^ | — | — | — | — | — | — |
| Model 3 | 135.330^***^ | 14 | 9.666 | 0.988 | 0.976 | 0.053 (0.045 0.061) |
| Model 4 | 122.156^***^ | 19 | 6.429 | 0.990 | 0.985 | 0.042 (0.035 0.049) |

Note.

^a^ Model 2 could not be properly identified when applied to the data at T1.

^***^*p* < 0.001.

**Table S3** Factor loadings of the competing models using the data at T1

| Item | Model 1 | |  | Model 3 | |  | Model 4 | |
| --- | --- | --- | --- | --- | --- | --- | --- | --- |
|  | DM | SC |  | DS | Neg |  | DS | Pos |
| n6 | 0.72 |  |  | 0.26 | 0.71 |  | 0.75 |  |
| n7 |  | 0.67 |  | 0.20 | 0.60 |  | 0.63 |  |
| n11 |  | 0.55 |  | 0.20 | 0.49 |  | 0.53 |  |
| n14 | 0.75 |  |  | 0.32 | 0.71 |  | 0.78 |  |
| n18 | 0.76 |  |  | 0.28 | 0.74 |  | 0.79 |  |
| n20 |  | 0.75 |  | 0.31 | 0.62 |  | 0.70 |  |
| p12 | 0.58 |  |  | 0.75 |  |  | 0.28 | 0.74 |
| p16 | 0.61 |  |  | 0.85 |  |  | 0.32 | 0.74 |
| AVE | 0.47 | 0.44 |  | 0.21 | 0.42 |  | 0.39 | 0.55 |
| Variance^a^ | 46.01% | |  | 53.12% | |  | 52.99% | |

Note.

^a^ The content in the last row demonstrated explained percentage of the factor(s) in each model to the total variance.

DM = Depressed Mood; SC = Somatic Complaints; DS = Depressive Symptoms; Neg = Negative Method; Pos = Positive Method; AVE = Average variance extracted.

**Table S4** Model fit indices of the structural invariance tests

| model | *χ²* | *df* | *χ²*/*df* | *Δχ²* | RMSEA (90% CI) | CFI | TLI | ΔRMSEA | ΔCFI |
| --- | --- | --- | --- | --- | --- | --- | --- | --- | --- |
| Invariance test across gender at T1 (N=3099, Male=1648, Female=1451) | | | | | | | | | |
| FVCV | 157.523^***^ | 47 | 3.352 | 10.409^*^ | 0.039 (0.032 0.046) | 0.989 | 0.987 | -0.002 | 0.000 |
| FMn | 207.895^***^ | 60 | 3.465 | 23.257^***^ | 0.040 (0.034 0.046) | 0.986 | 0.987 | 0.006 | -0.004 |
| Invariance test across gender at T2 (N=1978, Male=1027, Female=951) | | | | | | | | | |
| FVCV | 74.083^***^ | 47 | 1.576 | 2.319 | 0.024 (0.013 0.034) | 0.997 | 0.997 | -0.006 | 0.001 |
| FMn | 146.538^***^ | 60 | 2.442 | 24.998^***^ | 0.038 (0.030 0.046) | 0.991 | 0.991 | 0.016 | -0.006 |
| Invariance test over time (T1: N=3099; T2: N=1978) | | | | | | | | | |
| FVCV | 199.049^***^ | 99 | 2.011 | 2.804 | 0.018 (0.014 0.022) | 0.995 | 0.993 | -0.001 | 0.001 |
| FMn | 295.965^***^ | 112 | 2.643 | 29.201^***^ | 0.023 (0.020 0.026) | 0.990 | 0.989 | 0.003 | -0.003 |
| Longitudinal cross-gender invariance test (T1: N=3099, Male=1648, Female=1451; T2: N=1978, Male=1027, Female=951) | | | | | | | | | |
| FVCV | 458.200^***^ | 217 | 2.112 | 57.331^***^ | 0.027 (0.023 0.030) | 0.987 | 0.986 | 0.006 | -0.006 |
| FMn | 547.418^***^ | 254 | 2.155 | 62.329^***^ | 0.027 (0.024 0.030) | 0.984 | 0.985 | 0.006 | -0.007 |

Note. ^*^*p* < 0.05. ^***^*p* < 0.001.

FVCV = factor variance-covariance; FMn = factor means. Factor variance-covariance invariance models are nested in the metric invariance models; Factor means invariance models are nested in the threshold invariance model.

**Table S5** actual gender and temporal differences in the CES-D 8 factor scores

|  |  | Male/T1 | | Female/T2 | | Group difference | |
| --- | --- | --- | --- | --- | --- | --- | --- |
|  | factor | *M* | *SD* | *M* | *SD* | *t* | *df* |
| gender differences at T1 | NS | 0.04 | 0.80 | 0.16 | 0.95 | -3.93^***^ | 2845.96 |
|  | DHF | 0.03 | 0.81 | 0.00 | 0.72 | 0.93 | 3096.94 |
| gender differences at T2 | NS | 0.03 | 0.84 | 0.14 | 0.85 | -2.97^**^ | 1976 |
|  | DHF | 0.03 | 0.81 | -0.17 | 0.82 | 5.49^***^ | 1976 |
| temporal differences | NS | 0.04 | 0.91 | 0.16 | 0.85 | -7.23^***^ | 3098 |
|  | DHF | 0.03 | 0.99 | -0.07 | 0.85 | 5.60^***^ | 3098 |
| cross-gender temporal differences | NS_T2_- NS_T1_ | 0.05 | 0.85 | 0.12 | 0.91 | -2.14^*^ | 2987.45 |
|  | DHF_T2_-DHF _T1_ | -0.00 | 1.14 | -0.22 | 0.98 | 5.71^***^ | 3095.93 |

Note. ^*^*p* < 0.05. ^**^*p* < 0.01. ^***^*p* < 0.001.

NS = Negative Symptoms; DHF = Diminished Happiness Feeling.
